# Quantitative fluorescence endoscopy: an innovative endoscopy approach to evaluate neoadjuvant treatment response in locally advanced rectal cancer

**DOI:** 10.1136/gutjnl-2019-319755

**Published:** 2019-09-18

**Authors:** Jolien J J Tjalma, Marjory Koller, Matthijs D Linssen, Elmire Hartmans, Steven de Jongh, Annelies Jorritsma-Smit, Arend Karrenbeld, Elisabeth G de Vries, Jan H Kleibeuker, Jan Pieter Pennings, Klaas Havenga, Patrick HJH Hemmer, Geke AP Hospers, Boudewijn van Etten, Vasilis Ntziachristos, Gooitzen M van Dam, Dominic J Robinson, Wouter B Nagengast

**Affiliations:** 1 Department of Gastroenterology and Hepatology, University of Groningen, University Medical Center Groningen, Groningen, The Netherlands; 2 Department of Surgery, University of Groningen, University Medical Center Groningen, Groningen, The Netherlands; 3 Department of Clinical Pharmacy and Pharmacology, University of Groningen, University Medical Center Groningen, Groningen, The Netherlands; 4 Department of Pathology, University of Groningen, University Medical Center Groningen, Groningen, The Netherlands; 5 Department of Medical Oncology, University of Groningen, University Medical Center Groningen, Groningen, The Netherlands; 6 Department of Radiology, University of Groningen, University Medical Center Groningen, Groningen, The Netherlands; 7 Institute for Biological and Medical Imaging, Helmholtz Zentrum München, Munich, Germany; 8 Otolaryngology and Head and Neck Surgery, Erasmus MC, University Medical Center Rotterdam, Rotterdam, The Netherlands

**Keywords:** molecular oncology, colorectal cancer, colorectal surgery, imaging, fluorescence endoscopy

## Message

Quantitative fluorescence endoscopy (QFE) is a new technique that can visualise and quantify fluorescently tagged tumour tissue. In 25 patients with locally advanced rectal cancer (LARC), we evaluated QFE targeting vascular endothelial growth factor A (VEGFA) to detect residual tumour after neoadjuvant chemoradiotherapy (nCRT). QFE detected significantly higher fluorescence in tumour compared with normal rectal tissue and fibrosis, and improved prediction of final pathology results in 16% of patients compared with standard MRI and white-light endoscopy. QFE is a promising technique to aid clinical response assessment in patients with LARC and warrants further validation in larger clinical trials. ClinicalTrials.gov (NCT01972373).

## In more detail

**Figure 1:**
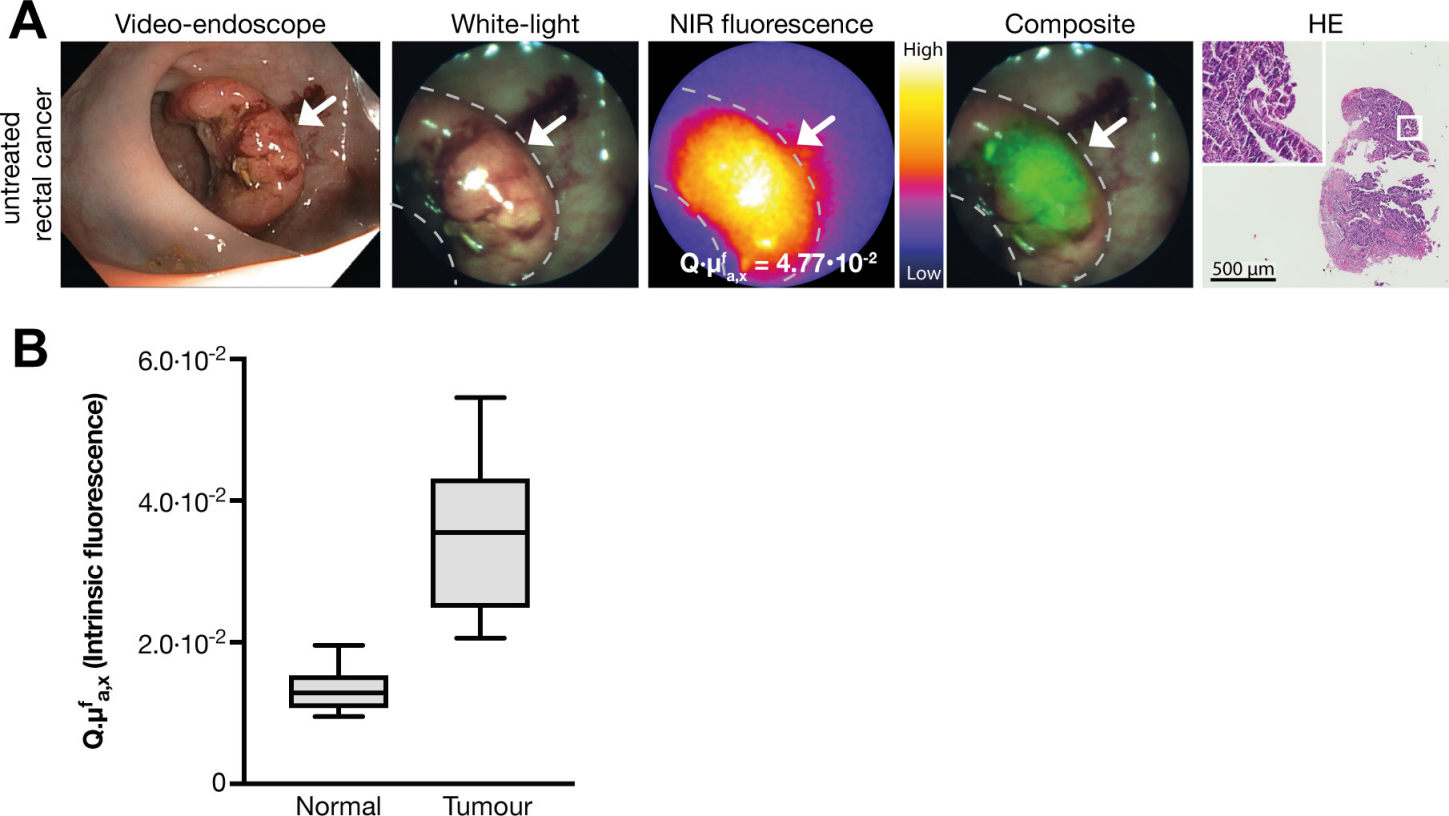
(A) Representative fluorescence images of the quantitative fluorescence endoscopy (QFE) procedure in untreated rectal cancer. From left to right: a high-definition white-light video endoscope image; a white-light image from the QFE fibreoptic, followed by the corresponding near-infrared (NIR) fluorescence image captured with an exposure time of 100 ms and the composite image of both modalities. The maximum quantified fluorescence value is depicted on the NIR fluorescence image. The rightmost image shows the HE staining of a forceps biopsy of the fluorescent area, confirming adenocarcinoma. (B) Fluorescence quantification results in 10 untreated patients. Tumour tissue shows higher fluorescence compared with normal rectal tissue. Boxplot centreline is at median, the bounds of the box at 25th to 75th percentiles, the whiskers depict the minimum–maximum.

Patients with LARC receive nCRT followed by surgery to achieve local disease control. Interestingly, 15%–27% of patients have a *pathological* complete response, that is, no residual cancer cells are found in the surgical specimen.^1–3^ There is an increasing interest in identifying patients with a *clinical* complete response before surgery, as non-operative management for these patients is associated with high survival rates, reduced morbidity and improved functional outcomes.^4–8^ However, assessing tumour response after nCRT is challenging. White-light endoscopy provides only morphological information, while MRI cannot always distinguish viable tumour from fibrosis.^9–11^ QFE is a novel endoscopy technique that visualises and quantitatively measures the presence of targeted fluorescence tracers in tissue. We hypothesised that VEGFA-targeted QFE can be of additional value in restaging patients with LARC. In untreated patients, QFE showed clearly enhanced fluorescence in all rectal tumours compared with normal rectal tissue (figure 1A). The tumour-to-normal ratio of 3.1 (figure 1B) signifies QFE can be used to localise rectal cancer.

**Figure 2:**
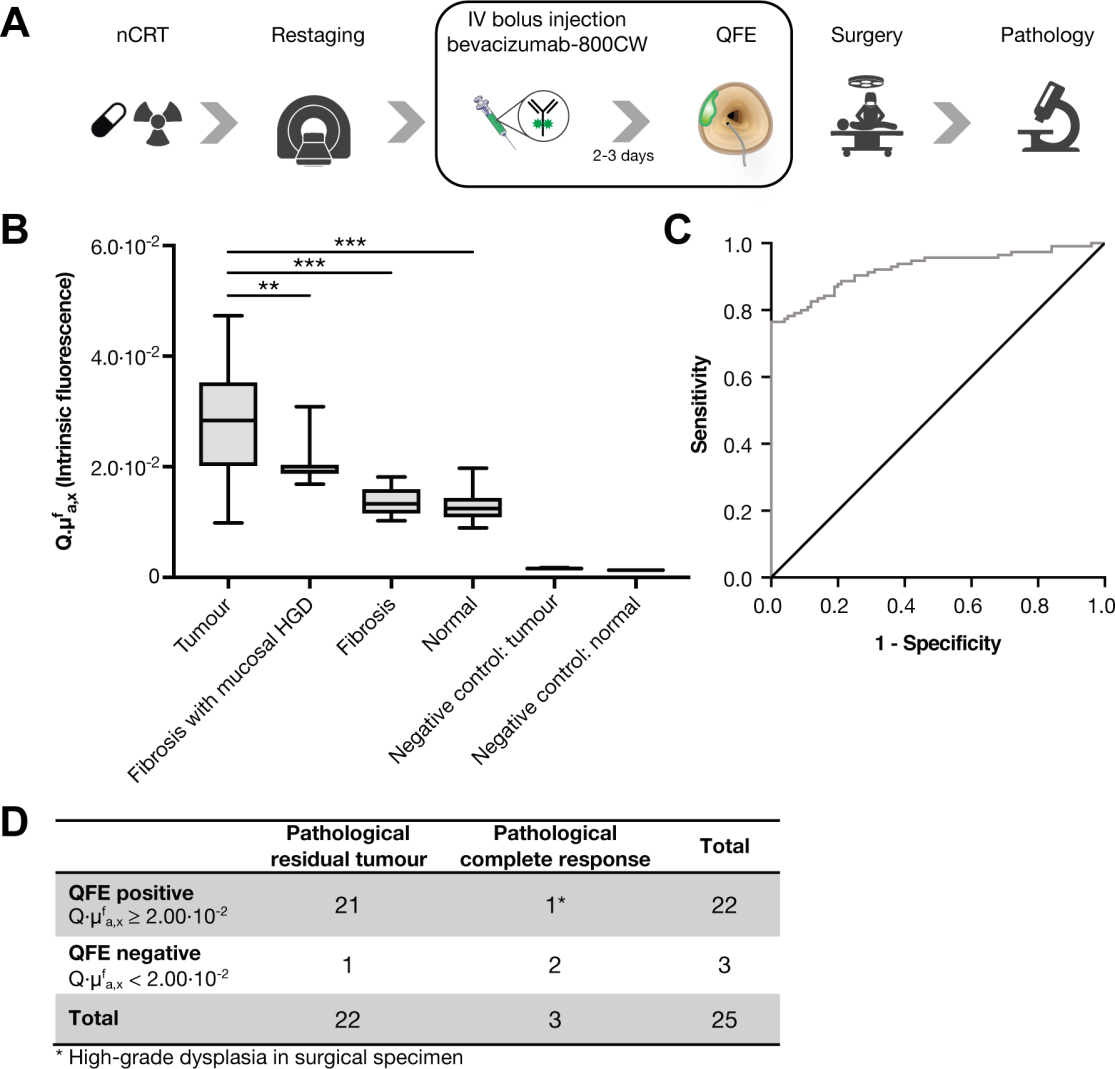
(A) Schematic overview of the clinical and study procedures. 4.5 mg bevacizumab-800CW was intravenously administered 2–3 days prior to quantitative fluorescence endoscopy (QFE). QFE consisted of wide-field fluorescence imaging, followed by fluorescence quantification using MDSFR/SFF spectroscopy and taking four forceps biopsies of normal rectal tissue (10 cm proximal from the tumour) and of 4 areas of the rectal tumour when present. (B) Fluorescence quantification results of the QFE procedures after neoadjuvant chemoradiotherapy (nCRT), depicted per tissue type. Tumour tissue shows higher fluorescence compared with fibrosis and normal tissue. Negative control tissue (of measurements of tumour and normal rectal tissue from a patient without tracer) showed no detectable fluorescence, signifying the measured fluorescence originated from the tracer. Boxplot centreline is at median, the bounds of the box at 25th to 75th percentiles, the whiskers depict the minimum–maximum. **p≤0.01; ***p≤0.001, one-way ANOVA test with Tukey post hoc analysis. (C) The receiver operating characteristic curve of quantified fluorescence of normal rectal tissue (n=100 measurements) vs tumour tissue (n=115 measurements) shows an area under the curve of 0.925. Normal rectal tissue included normal rectal tissue measurements of all patients and fibrosis measurements of pathological complete response. Tumour tissue included all lesion measurements of all patients with residual tumour at pathological examination. (D) Contingency table. HGD, high-grade dysplasia.

In this pilot study, we included 25 patients with LARC who were treated with nCRT (online supplementary table S1). QFE was performed at day of surgery, which enables comparison of QFE with standard clinical restaging (MRI and white-light endoscopy) and correlation to histopathology of the surgical specimen (figure 2A).

10.1136/gutjnl-2019-319755.supp3 Supplementary data

**Figure 3:**
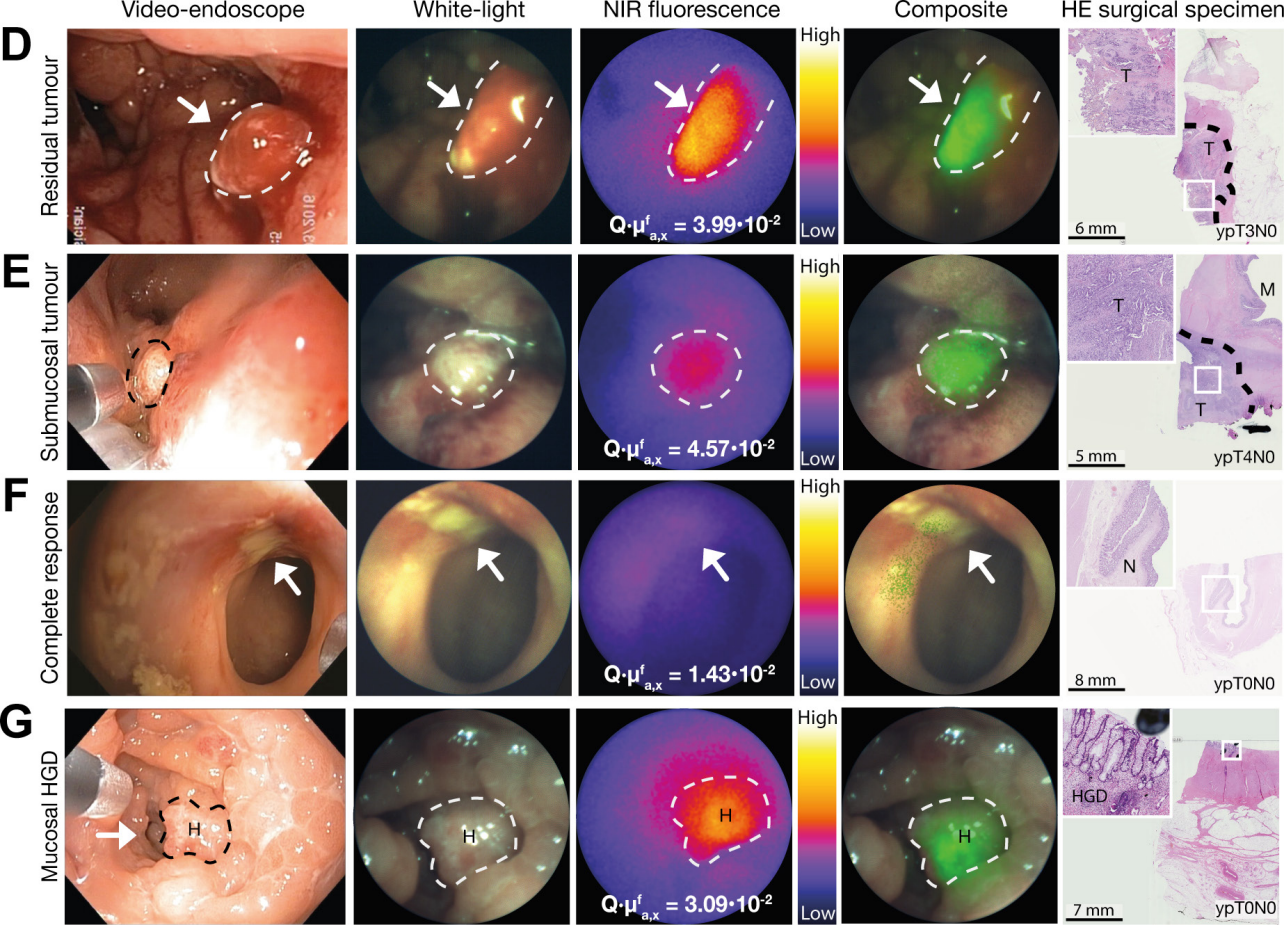
(A–D) Representative images of the quantitative fluorescence endoscopy (QFE) procedure after neoadjuvant chemoradiotherapy of a patient with (A) residual tumour, (B) submucosal tumour, (C) mucosal high-grade dysplasia (HGD) and (D) a pathological complete response. From left to right: a high-definition white-light video endoscope image of the rectal tumour; a white-light image from the QFE fibreoptic, followed by the corresponding near-infrared (NIR) fluorescence image captured with an exposure time of 100 ms and the composite image of both modalities. The maximum quantified fluorescence value is depicted on the NIR fluorescence image. The rightmost column depicts an HE staining of the surgical specimen in which the pathological TNM stage is indicated.

In all patients, vital tumour tissue showed high fluorescence compared with normal rectal tissue or fibrosis (online supplementary figure S1). Fluorescence quantification confirmed that fluorescence of tumour tissue (n=155 measurements) was higher than normal rectal tissue and fibrosis (n=100 measurements) (p<0.001) (figure 2B). The receiver operating characteristic curve showed a fluorescence cut-off value of 2.00×10^−2^ (area under the curve 0.925) (figure 2C, D). QFE was true positive in 21 of 25 patients as mucosal tumour (n=19, figure 3A) or even submucosal tumour (n=2, figure 3B) was confirmed by histology. QFE was truly negative in 2 of 25 patients, as histology confirmed pathological complete response (ypT0N0) (figure 3C). QFE was false positive in 1 of 25 patients, who showed extensive polypoid tissue on white-light endoscopy with one apparent fluorescent spot, where histology showed no invasive tumour (ypT0N0), but instead one locus with high-grade dysplasia (figure 3D). In 1 of 25 patients, QFE was false negative, and histology showed microscopic residual tumour: one locus situated in the submucosa.

10.1136/gutjnl-2019-319755.supp1 Supplementary data

Compared with standard clinical restaging, QFE would have changed the diagnosis in 4 of 25 patients (16%) (online supplementary figure S2). Three patients, categorised as clinical complete responders by MRI and white-light endoscopy, showed fluorescence with QFE and indeed showed vital tumour at histopathological examination (n=2) or regrowth already after 2 months of watchful waiting (n=1). One patient was clinically categorised as having residual tumour, but QFE showed low fluorescence and pathological examination confirmed a pathological complete response.

10.1136/gutjnl-2019-319755.supp2 Supplementary data

In our small sample size of 25 patients, the initial positive predictive value was 95% for QFE compared with 87.5% for MRI and 90% for white-light endoscopy. The accuracy of QFE was 92% compared with 84% for MRI and 80% for white-light endoscopy.

Find more details on online supplementary methods and results.

## Comments

This is the first-in-human study demonstrating that in vivo VEGFA-targeted QFE can improve the response assessment of patients with LARC after nCRT. We observed a sensitivity of 95% and accuracy of 92% for QFE compared with the reported respectively 71% and 89% of MRI combined with white-light endoscopy.^10^ The addition of QFE to MRI and white-light endoscopy resulted in more accurate clinical restaging in 16% of patients. Moreover, QFE is easy to perform during white-light endoscopy: the imaging and spectroscopy probes can be inserted in the working channel of any clinical video endoscope, the QFE measurements are operator independent and the procedure takes slightly more time (5–10 min). Importantly, no tracer-related or procedure-related adverse events were observed in this study.

When QFE is applied for restaging purposes, fluorescence quantification is important. Wide-field fluorescence visualisation alone does not necessarily reflect true tracer accumulation as fluorescence is influenced by tissue optical properties and could therefore lead to incorrect recommendations in clinical practice. By quantifying the fluorescence with multi-diameter single fibre reflection/single fibre fluorescence (MDSFR/SFF) spectroscopy, the fluorescence signals are corrected for tissue optical properties like scattering and absorption, circumventing this problem.^12 13^

Recent follow-up data showed that 19% of patients in watchful waiting, experience early tumour regrowth within 12 months.^14^ The majority of these patients had ypT3 or ypT4 disease at salvage, suggesting the presence of residual disease, intraluminal and also in deeper layers of the rectum. QFE might improve identification of these patients, as in this study QFE measured increased fluorescence in two of three patients with only submucosal tumour, that is, no tumour reaching the rectal mucosa. We hypothesise that bevacizumab-800CW could accumulate at the mucosal side because the tumour microenvironment was not yet normalised after nCRT, with still increased levels of VEGFA. A tracer that accumulates in the microenvironment could therefore offer an advantage for restaging compared with tracers that target proteins on tumour cell membranes. In addition, bevacizumab-800CW is a near-infrared tracer allowing deeper tissue penetration compared with tracers in the visible spectrum.

In this pilot study, QFE was false positive in one patient who turned out to have one locus of high-grade dysplasia at the rectal lumen. This is not surprising as a former study showed that bevacizumab-800CW also accumulates in low-grade and high-grade dysplastic adenomas which hampers discrimination between dysplasia and cancer.^13^ QFE was false negative in one patient who had one microscopic tumour locus present in the submucosa. Possibly, raising the tracer dose could provide stronger fluorescence signals and thus improve QFE detection. A clinical dose-finding study using bevacizumab-800CW for detection of colorectal adenomas reported that a higher tracer dose of 25 mg increased the target-to-background ratio almost twofold.^13^ Potentially, future complementary detection systems such as optoacoustic imaging, which combines the rich contrast of optical imaging with the higher penetration of radiofrequency waves, may further improve submucosal evaluation.

Our study has some limitations. We found a relatively low specificity and negative predictive value of QFE (67%) in this feasibility study, which might be due to the relatively small sample size. Next to this, the included patients were referred to our tertiary centre and represent patients with relatively complex LARC with extensive tumour (T4 in 40%) and high nodal stage (N2 in 64%) compared with the patients with relatively uncomplex LARC in standard practice. This also resulted in a relatively small portion of patients who experienced a pathological complete response (12%), compared with 15%–27% pathological complete response described in the literature.^1 2^

In conclusion, the results of this pilot study, even in this small group of patients, are encouraging and are potentially a first step towards quantitative fluorescence endoscopy for tumour response evaluation following neoadjuvant treatment. Ultimately, the combination of MRI, white-light endoscopy and QFE may prove to be the strategy to evaluate individual patient response and guide clinical decision-making. To realise this strategy, the capability of QFE in clinical response evaluation in patients with LARC, including determination of a definitive cut-off value that discriminates tumour from normal tissue, needs further evaluation in a larger prospective cohort.
