# Supplementary material for: Quantitative fluorescence endoscopy: an innovative endoscopy approach to evaluate neoadjuvant treatment response in locally advanced rectal cancer
Source: Gut. 2019 Sep 18;69(3):406–10. doi: 10.1136/gutjnl-2019-319755 (PMC7034345; doi:10.1136/gutjnl-2019-319755)
Supplement: Supplementary data [file gutjnl-2019-319755supp003.pdf]

SUPPLEMENTARY METHODOLOGICAL DETAILS

Summary of the study design

Patients with LARC were given a single dose of bevacizumab-800CW intravenously 2-3 days prior to the procedure. QFE was performed after the completion of nCRT, at day of surgery. In ten patients, QFE was also performed in untreated patients. Tracer uptake in LARC was compared to normal tissue in order to evaluate the detection of residual tumour and aid clinical response assessment after completion of nCRT. Patients also underwent conventional clinical restaging (MRI and white-light endoscopy). All results were compared to the gold standard: histopathological staging of the surgical specimen.

Study population

A total of 25 patients with proven LARC were enrolled between October 2013 and December 2016, in this non-blinded, prospective, single centre feasibility study. Patients were required to have histopathologically confirmed adenocarcinoma, with the lower margin within 16 cm from the anal verge. The pelvic MRI indicated at least one of the following criteria: cT4a, cT4b, N2, presence of tumour cells in the vasculature beyond the muscularis propria –extramural venous invasion (EMVI)–, presence of tumour or lymph node <1 mm from the mesorectal fascia (MRF) or positive lateral lymph nodes. Patients were eligible only if the multidisciplinary team decided on long-course nCRT. Patients were allowed also to be included in the RAPIDO trial (ClinicalTrials.gov Identifier NCT01558921). Key exclusion criteria were concurrent uncontrolled medical conditions and pregnancy or breast-feeding. Eligible patients were identified during the multidisciplinary colorectal cancer meeting at the

University Medical Centre Groningen (UMCG, Groningen, the Netherlands). All patients gave written informed consent for participation in the study before inclusion. The study protocol was approved by the Medical Ethics Committee of the UMCG and registered with ClinicalTrials.gov (NCT01972373).

Patient and Public Involvement

The study was supported by the Dutch Cancer Society. There was no patient involvement in study design, interpretation of results or writing of the manuscript.

Clinical procedures

Neoadjuvant treatment

Patients underwent nCRT before surgery, consisting of 28 doses of 1.8 Gy and oral capecitabine (825 mg/m^2 twice daily orally during radiotherapy course or 1000 mg/m^2 twice daily during 2 cycles of 14 days during radiotherapy course) or according to the study arm of the RAPIDO trial (n=2): 5 doses of 5 Gy, followed by 6 courses every 3 weeks of oxaliplatin intravenously (130 mg/m^2) at day 1 and oral capecitabine (1000 mg/m^2) twice daily for 14 days starting at day 1 of the course. Dose adjustments were made in the event of side effects. No post-operative chemotherapy was administered, in line with our national guideline.[1]

Clinical restaging

All patients underwent radiological restaging after nCRT, which consisted of a computer tomography (CT) scan of chest and abdomen and a diffusion-weighted MRI scan of the pelvis. Tumour (T), lymph node (N) and metastasis (M) stage were

assessed, together with EMVI and MRF, according to the TNM classification off the American Joint Committee on Cancer (5th edition). Endoscopic ultrasound was not used as standard clinical restaging modality.

Surgical resection

After the restaging CT and MRI, the surgical plan was formulated. Surgery consisted of abdominoperineal resection, low anterior resection or a more extended procedure like partial or full pelvic exenteration in order to reach a tumour-free circumferential resection margin. Although watchful waiting was not part of standard clinical care in our institution at the moment of the study, two patients requested this even though MRI and white-light endoscopy were inconclusive.

Pathological examination

Standard pathologic tumour staging of the resected specimen was performed by dedicated gastrointestinal cancer pathologist blinded for QFE results. The pathologic stage (ypTN) was recorded according to the fifth edition of the TNM classification, the clinical standard for the Netherlands. Circumferential resection margin involvement and lymphovascular invasion status were documented. pCR was defined as absence of viable adenocarcinoma cells in the surgical specimen (ypT0N0).

Study procedures

10 included untreated patients with LARC received a QFE procedure right after diagnosis, before nCRT. This was performed to verify if bevacizumab-800CW would accumulate in the rectal tumour tissue. Twenty-five patients received a QFE

procedure after nCRT, at day of surgery, enabling direct correlation with the current clinical standards: radiological restaging (cTNM), white-light video endoscopy and the pathological outcome of the surgical specimen (ypTNM).

Tracer production and administration

The monoclonal antibody bevacizumab (Roche, Hertfordshire, United Kingdom) was labelled under cGMP conditions with the near-infrared fluorophore IRDye800CW (IRDye800CW-NHS ester; LI-COR Biosciences, Lincoln, NE, USA) at the Department of Clinical Pharmacy and Pharmacology of the UMCG.[2] This was originally performed in a 4:1 dye-to-protein molar ratio. After the first six patients, the dye-to-protein molar ratio was changed to 2:1 to improve long term stability. No changes were seen in immunoreactivity tests. Patients received 4.5 mg of bevacizumab-800CW in accordance with microdosing limits as defined by the FDA.[3] Tracer was administered via intravenous bolus injection, 2 to 3 days prior to the QFE procedure, the optimal time-to-imaging interval based on experience with ^{89}Zr-bevacizumab PET-scans.[4] No tracer-related serious adverse events were reported, in accordance with previous clinical studies.[5-8]

White-light endoscopy procedure

All study subjects first received white-light endoscopy with a routine clinical high-definition video endoscope, immediately followed by QFE. Tumour response was endoscopically assessed by a dedicated gastroenterologist (W.B.N.) according to watchful waiting criteria: CR was diagnosed if residual tumour was absent, and only a flat, white scar with or without telangiectasia was present. Potential CR was

diagnosed when a small, flat ulcer with smooth edges without signs of residual polypoid tissue was present. Every other type of ulcer or mass was considered as definite residual tumour.[9]

QFE procedure

After high-definition white-light inspection of the rectum with a routine clinical high-definition video endoscope, the wide-field optical fibre was inserted through the working channel of the endoscope for wide-field fluorescence imaging. The gastroenterologist observed the presence, distribution and intensity of fluorescence signals in normal rectal tissue and in all rectal lesions present at endoscopy. Fluorescence was visually categorized as low (no difference with surrounding normal rectal tissue), intermediate (elevated, but difficult to clearly differentiate from surrounding normal rectal tissue) or high (clear differentiation from surrounding normal rectal tissue based on fluorescent signals). Images of normal tissue and LARC cancer tissue were digitally recorded with an exposure time of 1 frame per second and at video rate (10 frames per second). Subsequently, the spectroscopy fibre was inserted through the working channel of the endoscope and held onto tissue of interest, to perform *in vivo* point measurements for quantification of the NIR fluorescence. Quantification of minimal 3 different tumour areas and normal rectal mucosa was performed, preferably 10 cm proximal of the rectal tumour. At the end of the QFE procedure, four small forceps biopsies were taken of normal rectal tissue and of every tumour location where quantification was performed. *Ex vivo* spectroscopy measurements were performed on these fresh biopsies to enable direct correlation of NIR fluorescence with histopathology. As there were no significant

differences between the in vivo and ex vivo spectroscopy measurements, the measurements depicted in the result section were grouped. Afterwards, the tissue biopsies were formalin-fixed and paraffin-embedded (FFPE) or snap-frozen in liquid nitrogen and stored at -80° Celsius.

QFE system

Wide-field fluorescence imaging was provided by an imaging platform (SurgVision BV, Groningen, the Netherlands) consisting of an optical fibre-bundle coupled to a charge-coupled digital (EM-CCD) camera, sensitive for NIR light, and a separate camera for colour detection, as described previously.[7,8] Fluorescence excitation was provided by two class IIIb lasers (750 nm); white-light was provided by a LED light source. The wide-field fibre images (colour, fluorescence and composite) were displayed live on a separate monitor for the gastroenterologist.

Fluorescence quantification was performed with a Multi Diameter Single Fibre Reflectance and Single Fibre Fluorescence (MDSFR/SFF) spectroscopy device. The *in vivo* measurements were executed with a fibre-bundle consisting of two concentric rings, the *ex vivo* measurements were performed with a different fibre-bundle consisting of 2 adjacent fibres (0.4 and 0.8 mm). During a measurement, two consecutive reflection spectra were acquired from which the tissue light absorbance and light reflection were calculated.[10-12] This was immediately followed by a fluorescence spectrum measurement. The intrinsic fluorescence ($Q.\mu^{f}_{a,x}$) of bevacizumab-800CW was acquired by correcting the fluorescence spectrum for the calculated tissue optical properties.[10,13,14] We calculated the local tracer concentration based on the *in vivo* quantified fluorescence, the molar extinction of

the tracer and the fluorescence quantum yield.[8] In 6 patients, all with clear fluorescent endoluminal tumour present, the fluorescence quantification could not be performed due to malfunction of the spectroscopy device (lamp broken).

Correlation of QFE findings with radiological and pathological staging

To assess the value of QFE after nCRT, QFE findings were compared to the clinical restaging findings (MRI and high-definition white-light endoscopy) and correlated to the gold standard: pathological staging (ypTNM). The haematoxylin and eosin (HE) staining was performed on 4 μm FFPE tissue sections as standard clinical staining by our Pathology Department.

Statistical methods

Descriptive statistics were generated to describe patient characteristics and the association between QFE and pathological outcome. The intrinsic fluorescence ($Q{\cdot}\mu^f_{a,x}$) measurements of different tissue types after nCRT was analysed with a one-way ANOVA test with Tukey post-hoc analysis. A ROC curve was generated from all fluorescence measurements obtained from normal rectal tissue versus tumour tissue. Normal rectal tissue included normal rectal tissue measurements of all patients and fibrosis measurements of pathological complete responders. Tumour tissue included all lesion measurements of all patients with residual tumour at pathological examination. The median and maximum values of the intrinsic fluorescence measurements ($Q{\cdot}\mu^f_{a,x}$) were correlated, showing a good correlation (R^2 = 0.84, $P<0.0001$, data not shown). P values lower than 0.05 were regarded as statistically significant. IBM SPSS Statistics, version 23.0 (SPSS inc.) was used for all statistical

analyses. All authors had access to the study data and reviewed and approved the final manuscript.

SUPPLEMENTARY RESULTS

Patient characteristics

Twenty-five patients diagnosed with LARC were enrolled in the study. Ten of these patients received a baseline QFE prior to nCRT (untreated cancer), and all 25 patients received QFE after nCRT (table 1).

Supplementary Table S1 Patient and tumour characteristics

Characteristic	No.	%
Median age, in years (range)	61 (31-76)	
Sex		
Male	15	60%
**Female**	10	40%
Endoscopic findings at time of diagnosis		
**Non-passable stenosis**	*7*	*28%*
Radiologic staging (MRI pelvis and CT chest+abdomen)		
***c*T3 N0**	2	8%
***c*T3 N1**	5	20%
***c*T3 N2**	10	40%
***c*T4 N1**	4	16%
***c*T4 N2**	4	16%
Neoadjuvant chemoradiotherapy regimen		
Capecitabine 825 mg/m^2 **bid day 1-28 + 28x1.8Gy radiotherapy**	18	72%
**Capecitabine 1000 mg/m^2 bid day 1-14 and 25-38 + 25x2Gy radiotherapy**	5	20%

**6 cyles of capecitabine/oxaliplatin + 5x5Gy radiotherapy**	2	8%
Main endoscopic findings at restaging		
Residual tumour **/ polypoid tissue**	19	76%
**Ulcer >3 cm**	2	8%
**Ulcer <3 cm**	3	12%
**White-scar tissue**	1	4%
Type of Surgery		
Low anterior resection	14	56%
**Abdominoperineal resection**	11	44%
Pathological staging		
***yp*T0 N0 (pCR)**	3	12%
***yp*T2 N0**	4	16%
***yp*T3 N0**	6	24%
***yp*T3 N1**	3	12%
***yp*T3 N2**	6	24%
***yp*T3 N0 M1**	1	4%
***yp*T4 N0**	2	8%

REFERENCES

1 Breugom AJ, Swets M, Bosset J-F, et al. Adjuvant chemotherapy after preoperative (chemo)radiotherapy and surgery for patients with rectal cancer: a systematic review and meta-analysis of individual patient data. *Lancet Oncol* 2015;16:200–7.

2 Weele ter EJ, Terwisscha van Scheltinga AGT, Linssen MD, et al. Development, preclinical safety, formulation, and stability of clinical grade bevacizumab-

800CW, a new near infrared fluorescent imaging agent for first in human use. *Eur J Pharm Biopharm* 2016;104:226–34.

3 Lappin G. The expanding utility of microdosing. *Clin Pharmacol Drug Dev* 2015;4:401–6.

4 Oosting SF, Brouwers AH, van Es SC, et al. 89Zr-bevacizumab PET visualizes heterogeneous tracer accumulation in tumor lesions of renal cell carcinoma patients and differential effects of antiangiogenic treatment. *J Nucl Med* 2015;56:63–9.

5 Lamberts LE, Koch M, de Jong JS, et al. Tumor-specific uptake of fluorescent bevacizumab-irdye800CW microdosing in patients with primary breast cancer: a phase I feasibility study. *Clin Cancer Res* 2017;23:2730–41.

6 Harlaar NJ, Koller M, de Jongh SJ, et al. Molecular fluorescence-guided surgery of peritoneal carcinomatosis of colorectal origin: a single-center feasibility study. *Lancet Gastroenterol Hepatol* 2016;1:283–90.

7 Nagengast WB, Hartmans E, Garcia-Allende PB, et al. Near-infrared fluorescence molecular endoscopy detects dysplastic oesophageal lesions using topical and systemic tracer of vascular endothelial growth factor A. *Gut* 2019;68:7-10.

8 Hartmans E, Tjalma JJJ, Linssen MD, et al. Potential red-flag identification of colorectal adenomas with wide-field fluorescence molecular endoscopy. *Theranostics* 2018;8:1458–67.

9 Maas M, Lambregts DMJ, Nelemans PJ, et al. Assessment of clinical complete response after chemoradiation for rectal cancer with digital rectal examination, endoscopy, and MRI: selection for organ-saving treatment. *Ann Surg Oncol* 2015;22:3873–80.

10 van Leeuwen-van Zaane F, Gamm UA, van Driel PBAA, et al. In vivo quantification of the scattering properties of tissue using multi-diameter single fiber reflectance spectroscopy. *Biomed Opt Express* 2013;4:696–708.

11 Gamm UA, Kanick SC, Sterenborg HJ, et al. Measurements of tissue scattering properties using multi-diameter single fiber reflectance spectroscopy: in silico sensitivity analysis. *Biomed Opt Express* 2011;2:3150-66.

12 van Leeuwen-van Zaane F, Gamm UA, van Driel PBAA, et al. Intrinsic photosensitizer fluorescence measured using multi-diameter single-fiber spectroscopy in vivo. *J Biomed Opt* 2014;19:1560–2281.

13 Middelburg TA, Hoy CL, Neumann HAM, et al. Correction for tissue optical properties enables quantitative skin fluorescence measurements using multi-diameter single fiber reflectance spectroscopy. *J Dermatol Sci* 2015;79:64–73.

14 Hoy CL, Gamm UA, Sterenborg HJCM, et al. Method for rapid multidiameter single-fiber reflectance and fluorescence spectroscopy through a fiber bundle. *J Biomed Opt* 2013;18:1560–2281.
