# Supplementary figures and images for: Quantitative fluorescence endoscopy: an innovative endoscopy approach to evaluate neoadjuvant treatment response in locally advanced rectal cancer
Source: Gut. 2019 Sep 18;69(3):406–10. doi: 10.1136/gutjnl-2019-319755 (PMC7034345; doi:10.1136/gutjnl-2019-319755)

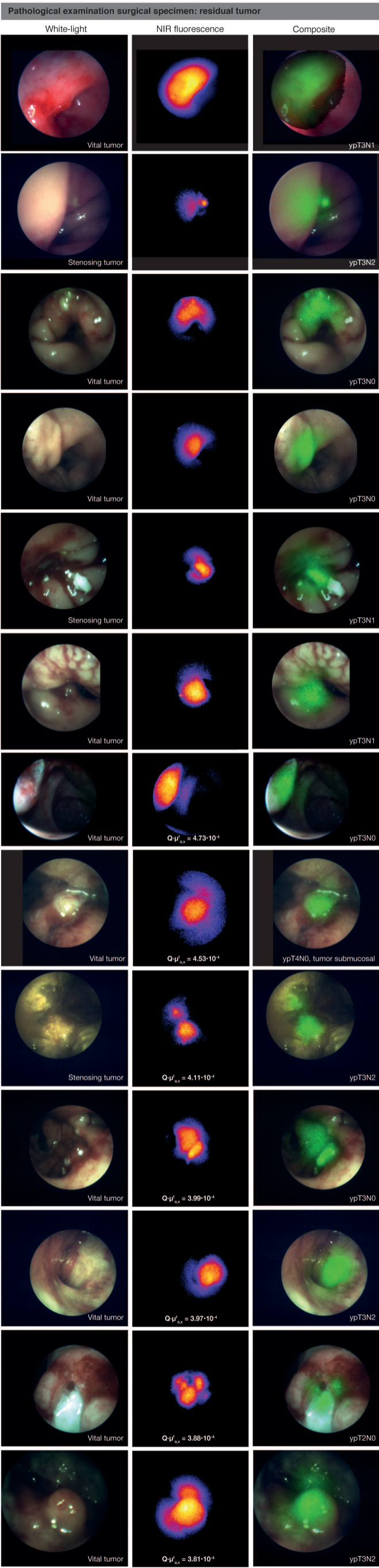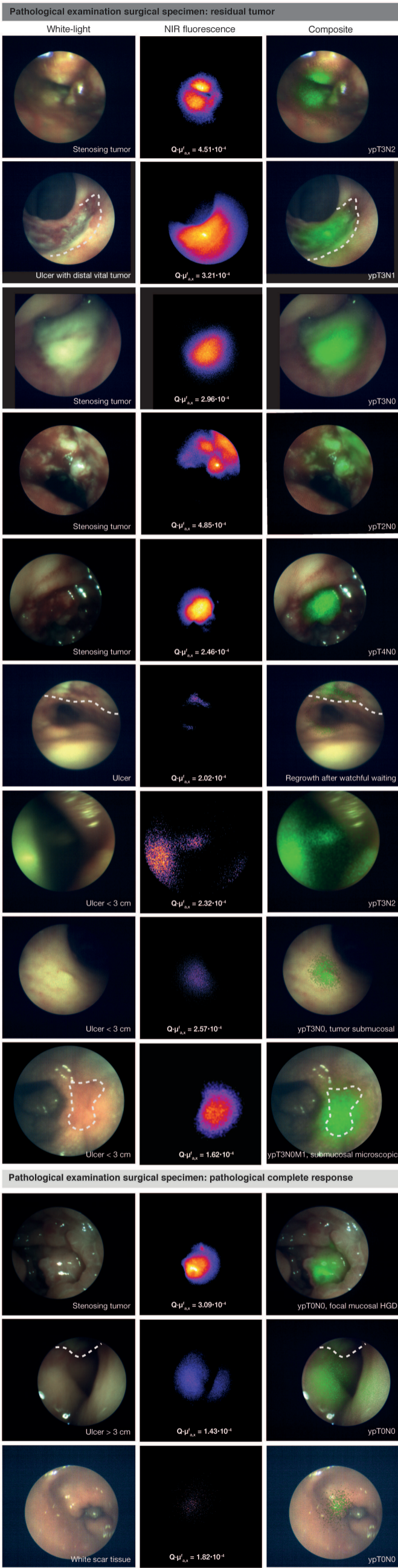

Supplement: Supplementary data [file gutjnl-2019-319755supp001.pdf]

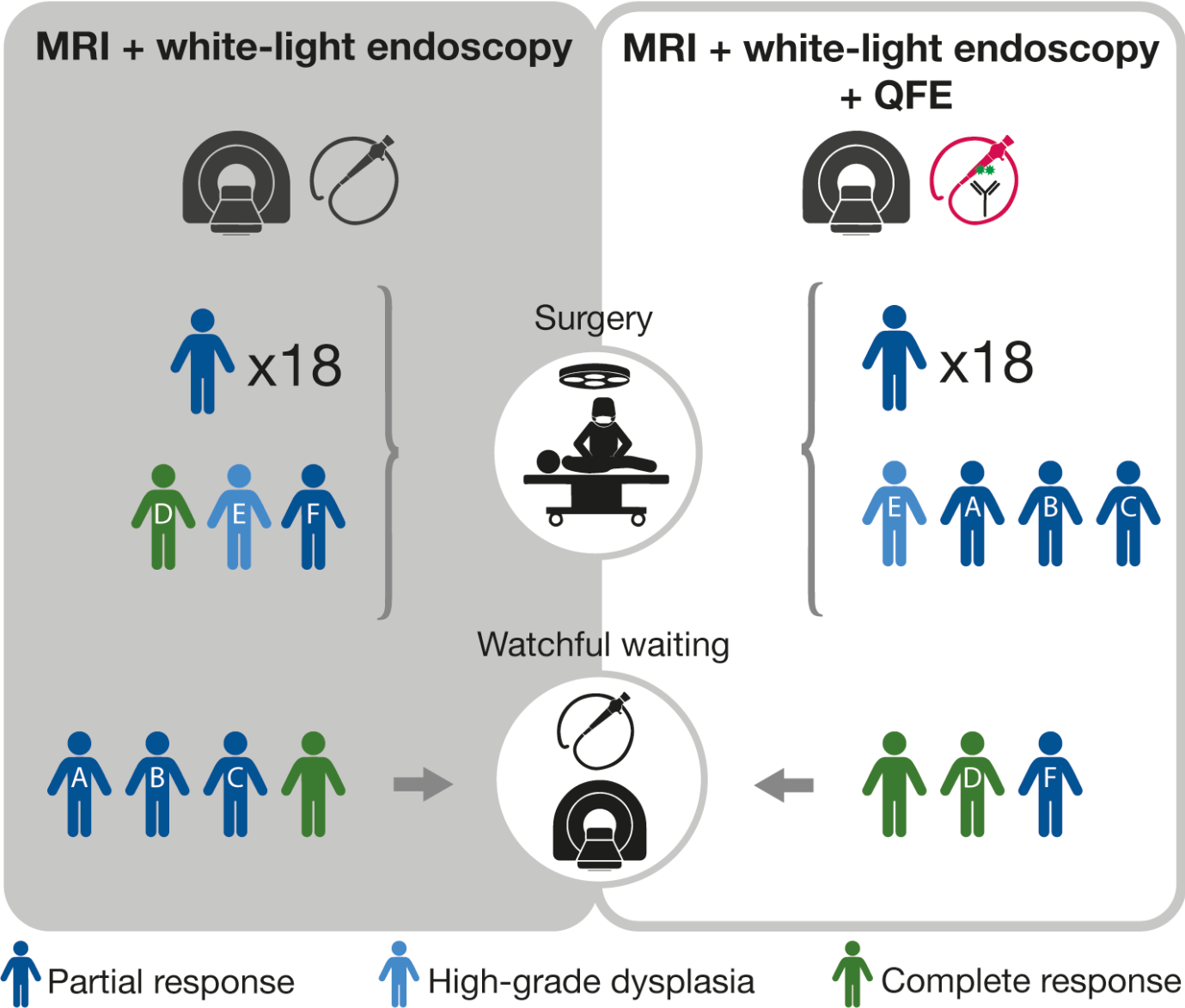

Supplement: Supplementary data [file gutjnl-2019-319755supp002.pdf]
